# The Impact of Glycosyl-Phosphatidyl-Inositol Anchored MICA Alleles on Novel NKG2D-Based Therapies

**DOI:** 10.3389/fimmu.2015.00193

**Published:** 2015-04-27

**Authors:** Mar Valés-Gómez

**Affiliations:** ^1^Department of Immunology and Oncology, National Centre for Biotechnology (CNB-CSIC), Madrid, Spain

**Keywords:** NKG2D, NKG2D-ligands, MICA/B, ULBP, GPI-anchored proteins, immune evasion, HCMV, exosomes

NKG2D is an activating receptor present in all human NK cells, CD8+ T cells, and in certain populations of CD4 T cells, and engagement of this receptor with its ligands is a crucial step in the regulation of both innate and specific immune responses [for review, see Ref. (1)]. NKG2D recognizes two families of MHC-related molecules, whose expression is, in general, upregulated when the cells suffer different types of stress including infection and tumorigenesis. In fact, a large variety of primary tumors and tumor-derived cell lines express NKG2D-ligands and, in the last few years, many reports have established correlations between the presence of NKG2D-ligands in cancer patient serum and disease prognosis [for review, see Ref. (2)]. The presence of NKG2D-ligands in serum is related to another important feature of these molecules: they can be released to the supernatant by either metalloprotease cleavage or incorporated in nanovesicles (exosomes), depending on their biochemical features [for review, see Ref. (3)]. However, it is still not understood why NKG2D-ligands are, in some instances, secreted while in other cases they remain at the cell surface, and understanding the cellular “decisions” on the pathways followed by these molecules will be of crucial importance in the choice of novel therapies based on NKG2D, such as monoclonal antibodies or NKG2D-CARs [for review, see Ref. (4)], since soluble ligands could interfere with these therapies in different manners depending on their biological form. In this sense, detailed study of the differential features of each individual NKG2D-ligand is still needed and investigation of the mechanisms used by different viral gene products to interfere with NKG2D-ligand expression will likely be critical in uncovering these cell biology issues.

## Human NKG2D-Ligands and Immune Regulation

**Figure 1:**
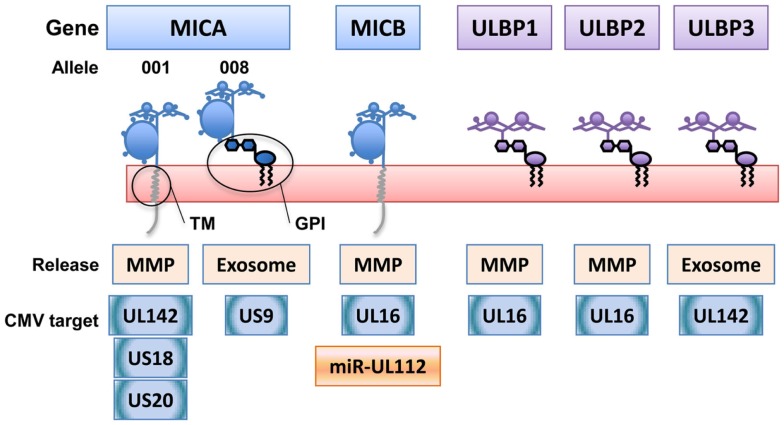
**NKG2D-ligands belong to two genetic families with multiple biochemical and cell biology features**. The two genetic families of NKG2D-ligands, MICA/B (major histocompatibility complex class I-related chain) and ULBPs (UL16-binding proteins), encode proteins with different membrane attachments [transmembrane (TM) and glycosyl-phosphatidyl- inositol (GPI)] and different mechanisms for release to the extracellular milieu [exosomes versus metalloprotease (MMP) cleavage]. However, not all the members of one of these genetic families of NKG2D-ligands share the same biochemical characteristics. NKG2D-ligands are also differentially recognized by viral proteins and miRNAs that interfere with the immune response. As an example, the human cytomegalovirus evasion molecules targeting NKG2D-ligands (proteins in blue, miRNA in orange) are depicted.

NKG2D-ligands belong to two genetic families with multiple members: the major histocompatibility complex class I-related chain (MIC)A/B and UL16 binding protein (ULBP) one to six molecules, also known as retinoic acid early transcripts (RAETs) [for review, see Ref. (5)]. Although both families of NKG2D-ligands are related to MHC molecules and their expression is increased after stress, many differences are observed in their biochemical and cell trafficking properties. Moreover, individual ULBP molecules and MICA/B alleles can share functional characteristics that are not generally conserved within their genetic family. For example, most MICA/B alleles are transmembrane proteins whereas ULBPs have glycosyl-phosphatidyl-inositol (GPI) anchors. However, MICA is a highly polymorphic gene and some of the most frequent alleles, the group known as MICA5.1 whose principal component is MICA*008, contain a frame-shift mutation in the transmembrane region that introduces an early stop codon. These changes mean that MICA5.1 alleles attach to the plasma membrane via a GPI anchor (6), like most of the ULBPs (Figure 1). MICA*008 also differs from other MICA alleles in that it has an extremely slow maturation rate (6, 7) and differs in the N-glycosylation requirements for cell trafficking (8). Functionally, the difference in membrane anchoring of MICA*008 alters its mechanism of release to the extracellular milieu and affects the modulation of the immune response, since multimeric, exosomal MICA*008 are more potent downmodulators of the NKG2D receptor than soluble monomeric MICA molecules (9) and could be more immunogenic (10). Thus, it is important to study the biology of individual NKG2D-ligands separately to understand their involvement in immune recognition and evasion, especially since the use of drugs to modulate immune responses, by regulating NKG2D-ligand release, could also have different outcomes depending on the release mechanism; for example, inhibition of metalloproteases leads to incorporation into exosomes of certain NKG2D-ligands (11).

## Study of Viral Immune Evasion Strategies Can Aid Understanding the Heterogeneity in the Cell Biology of NKG2D-Ligands

A striking feature of the biology of the MICA*008 allele is its resistance to most of the described viral immune evasion strategies. Human cytomegalovirus (HCMV), for example, encodes several viral proteins and miRNAs that interfere with NKG2D-ligand expression [(12–14) and references therein], yet MICA*008, was resistant to the MICA binding viral protein UL142 (15, 16), and was not downregulated upon infection with HCMV strain AD169*VarS* (17, 18). Indeed, MICA*008 has been considered to be an HCMV-resistant “escape variant,” and the high prevalence of this allele in multiple populations has been suggested to be the result of an advantage to human NK cells in recognizing infected cells. Recently, however, it has been shown that the US9 gene product of HCMV can selectively downregulate MICA*008 expression in a mechanism that depends on specific interference with the particular cell biology of this MICA allele (7). This observation exemplifies how detailed knowledge of the biochemistry of individual NKG2D-ligands can explain specific functional interactions of these molecules. US9 retention occurs in the ER compartment, in which the MICA*008 protein would be transferred to the GPI moiety (7), while UL142 retains MICA in the Golgi apparatus (16), suggesting different molecular requirements. In fact, UL142 retains inside the cell both the transmembrane MICA alleles and the GPI-anchored protein ULBP3. The use of cell lines susceptible to HCMV infection, but deficient in GPI-transferase enzymes (19, 20), could help to identify whether the GPI anchor is necessary for MICA*008 downregulation by US9 or the molecular mechanism for retention depends on another part of the molecule. Moreover, the information on viral escape and cellular biology of NKG2D-ligands could be applied in other systems in which we still need to understand the role of these molecules, mainly cancer.

## Toward Therapies Based on NKG2D Receptor and its Ligands: The Need for Personalized Medicine

That MICA*008 has different biochemical features to transmembrane MICA alleles leads to at least three important consequences: MICA*008 is released preferentially in exosomes, it has a very slow maturation process, and it is not affected by other MICA-modulating pathogen molecules (proteins or miRNAs). It is entirely plausible that in stressed systems, like cancer cells or autoimmunity, certain cellular routes could be blocked while other routes could be enhanced leading to alterations in intracellular protein trafficking. In such a case, NKG2D-ligands could change their expression from intracellular to secreted or appear at the plasma membrane, and this effect will in turn vary depending on the particular ligand. Therefore, it would not be advisable to treat all MICA alleles as biologically equivalent. To interfere intelligently with NKG2D-ligand expression, or to interpret adequately the significance of their expression, will require specific evaluation for each individual patient, determining MICA typing, the amount of ligands released to serum, and the levels of NKG2D receptor on their immune cells. More research is needed, in parallel, to better characterize more differences among MICA alleles and other ligands for NKG2D. In the meantime, it may be worthwhile to revisit the results of previous clinical assays adjusting the data to take into account the MICA typing of the patients, since it might be better to evaluate MICA behavior in function of transmembrane versus GPI-anchored.

## Conflict of Interest Statement

The research was conducted in the absence of any commercial or financial relationships that could be construed as a potential conflict of interest.
